# *Clostridioides difficile* spore: coat assembly and formation

**DOI:** 10.1080/22221751.2022.2119168

**Published:** 2022-09-29

**Authors:** Ji Zeng, Hao Wang, Min Dong, Guo-Bao Tian

**Affiliations:** a School of Biomedical and Pharmaceutical Sciences, Guangdong University of Technology, Guangzhou, People’s Republic of China; b Department of Microbiology, Harvard Medical School, Boston, MA, USA; c Department of Urology, Boston Children’s Hospital, Harvard Medical School, Boston, MA, USA; d Department of Microbiology, Zhongshan School of Medicine, Sun Yat-sen University, Guangdong, People’s Republic of China; e Advanced Medical Technology Center, The First Affiliated Hospital, Zhongshan School of Medicine, Sun Yat-sen University, Guangzhou, People’s Republic of China; f Key Laboratory of Tropical Diseases Control (Sun Yat-sen University), Ministry of Education, Guangzhou, People’s Republic of China; g School of Medicine, Xizang Minzu University, Xianyang, People’s Republic of China

**Keywords:** *Clostridioides difficile*, sporulation, spore formation, coat assembly, morphogenetic protein

## Abstract

*Clostridioides difficile* (*C. difficile*) is a Gram-positive, spore-forming, toxin-producing, obligate anaerobic bacterium. *C. difficile* infection (CDI) is the leading cause of healthcare-associated infective diarrhoea. The infection is mediated by the spore, a metabolically inactive form of *C. difficile*. The spore coat acts as a physical barrier to defend against chemical insults from hosts and natural environments. The composition of spore coat has already been revealed; therefore, the interactive networks of spore coat proteins and the dynamic process of coat assembly are the keys to design strategies to control and cure CDI. This review gives a brief discussion of the signal processing and transcriptional regulation of *C. difficile* sporulation initiation. Following the discussion, the spore formation is also introduced. Finally, this review mainly focuses on the spore coat assembly, a poorly understood process in *C. difficile*, and important proteins that have been studied.

## Introduction

*Clostridioides difficile* has been listed by the Centers for Disease Control and Prevention (CDC) as an urgent threat of antibiotic resistance, which causes about 0.25–0.5 million infection cases in America annually, including 13,000–20,000 in-hospital deaths [1–3]. The most effective treatment for CDI is still antibiotics; however, about 15–25% of patients with the first episode of antibiotics would have a relapse, and about 40–65% of patients with the first relapse would have further recurrences [4,5]. The virulence of CDI has mainly resulted from two large clostridial toxins, toxin A (TcdA) and toxin B (TcdB), and, in some strains, the third binary toxin CDT, the hypervirulent strain ribotype 027 (NAP1/B1/027), for example [2,6,7]. However, spores are the major transmission particles, which could endure heating, UV irradiation, and various chemical insults [4]. In addition, the persistence and recurrence of CDI are also suggested to be mediated by spores, which could enter into the intestinal epithelial cells as a reservoir for re-infection [3,8,9].

The first line of treatment for CDI is antibiotics (vancomycin, metronidazole, and fidaxomicin) [2,5]. However, antibiotics disrupt the gut microbiota and potentially increase CDI recurrence. Immune-based therapies against *C. difficile* toxins, such as vaccines and antibodies, are in clinical trials [10]. Yet, Bezlotoxumab, a human monoclonal antibody, is proven to be less effective against the hypervirulent clade TcdB2 due to sequence variation [11]. Toxin-based vaccine therapies may suffer the same difficulties [12]. Fecal microbiota transplantation seems to be an efficient treatment for recurrent *C. difficile* infection; however, the bio-safety of this method is still under debate [13]. Therefore, a safer and better cure for *C. difficile* infection is still in need, especially for recurrent CDI.

The spore coat plays a key role in resistance to chemical insults from hosts and natural environments during *C. difficile* infection. Therefore, understanding the dynamic process of coat assembly is the key to design strategies to control and cure CDI. A few reviews have been focused on spore formation and regulation [8,14–17]. However, important questions about the spore coat assembly remain elusive, such as the composition of each layer, the interactive network of spore coat proteins, the timing and order of protein attachment and encasement, etc. Therefore, this review summarizes our current understanding of the spore coat assembly and formation and hopefully would provide insights into the cure and control of *C. difficile* infection.

## Sporulation initiation regulation

Endospore is produced in Firmicutes and has been extensively studied in the model organism *Bacillus subtilis* [18]. The *C. difficile* spore is composed of a highly dehydrated core, a cortex layer composed of modified peptidoglycan, an orderly recruited proteinaceous spore coat layer, and an exosporium layer from inside to outside [8,19]. Sporulation initiation is a complex decision by integrating environmental signals and nutrient states, which are regulated by multiple factors and reviewed elsewhere comprehensively [14,20,21]. Therefore, we only give a brief discussion of a few important factors involved in the decision-making.

In *B. subtilis*, sensor histidine kinases (KinA, KinB, and KinC) transfer phosphate to the sporulation master regulator, Spo0A, via phosphorelay [22,23]. The Spo0A is conserved in *C. difficile*, even though the phosphorelay mechanism seems to be missing and the histidine kinases are yet to be identified [24,25]. Phosphorylation activates Spo0A, which, in turn, up-regulates early-sporulation genes to initiate the sporulation process [26–28]. Similar to its homologue in *B. subtilis*, Spo0A in *C. difficile* is composed of N-terminal phosphorylation and dimerization domain, and a C-terminal DNA-binding domain [27]. Additionally, it recognizes a similar binding motif, even though differences exist for the roles it plays during sporulation compared to *B. subtilis* [20,27].

SigH is a key regulator of the transition phase and sporulation initiation in *C difficile* [14,15]. It recognizes a similar motif to that in *B. subtilis*, and regulates genes involved in various bacterial activities and metabolisms, including sporulation, cellular division, motility, virulence, etc. [14,29] Spo0A and SigH reciprocally up-regulate each other, creating positive feedback to reinforce the activation [14].

Another regulator involved in *C. difficile* sporulation initiation is RstA, an RRNP family member [30]. RstA shares similarities with the *Bacillus* Rap proteins, containing an N-terminal Helix-Turn-Helix (HTH) DNA-binding motif, and several C-terminal tetratricopeptide repeat (TPR) domains [30]. RstA positively affects sporulation, possibly through the C-terminal TPR domain, and negatively affects toxin production, possibly through the N-terminal DNA-binding domain, though both effects are strain-dependent [30–32].

CodY is a transcriptional regulator that senses the metabolic state of the cell, which has been studied extensively in *B. subtilis* [33–36]. In a nutrient-rich state, CodY is bound by branched-chain amino acids (BCAAs) and GTP and represses nutrient acquisition and amino acid synthesis [34,35]. On the contrary, in a nutrient-limited state, the repression is alleviated by CodY which is no longer bound by its effectors. Similarly, CodY is a nutritional regulator in *C. difficile* [36]. The sporulation of *C. difficile* is negatively regulated by CodY, probably through the CodY-regulated *opp* and *sinR* operons, though the reduction of sporulation frequency is strain-specific [36].

Another regulatory protein involved in sporulation initiation is carbon catabolite protein A (CcpA), a member of the LacI/GalR family [37,38]. CcpA is the global regulator of carbon catabolite repression (CCR), regulating several hundred catabolic genes in *B. subtilis* [38]. Similarly, ∼140 genes are regulated by CcpA in *C. difficile* [37]. Spo0A and SigF, a sporulation-specific sigma factor, are negatively regulated by CcpA, through direct binding to their promoters [37]. Consistently, the sporulation in *C. difficile* JIR8094 strain increases significantly (1–2 magnitude) in *ccpA* mutant at 24 h. However, the repression of sporulation by CcpA seems to be irrelevant to glucose, such that the sporulation efficiencies of JIR8094 and *ccpA* mutant are similar at 48 and 96 h, and both are strongly reduced in the presence of glucose [37].

Sporulation initiation is regulated by a complex regulatory network with Spo0A as the master regulator. Many factors have been identified; however, the coordination and/or competition effects of these regulators through Spo0A are yet to be clarified. Moreover, it is quite possible that more regulators, especially activating factors, are yet to be identified, considering direct activators of Spo0A are still missing [14]. The phosphorylation level of Spo0A defines the developmental pathways, sliding, biofilm formation, or sporulation, in *B. subtilis* [39]. Therefore, it is intriguing to characterize the roles of Spo0A, or other regulators, in decision-making of vegetative cells, toxin production, sporulation, or biofilm, in *C. difficile*. Last but not the least, the sporulation frequency of *C. difficile* is strain-specific, and few clinical isolates couldn’t produce spores quantified by *in vitro* assays [3,40–42]. It has been reported that sporulation frequency, but not ribotype, is correlated with the clinical severity and recurrence of CDI [40–42]. It is known that clinical outcomes are affected by multi-factors, such as toxigenicity, antibiotic resistance, spore germination, host microbiota, etc. [43]. Nevertheless, the real contribution of sporulation is a small but essential question.

## Spore formation

**Figure 1.**
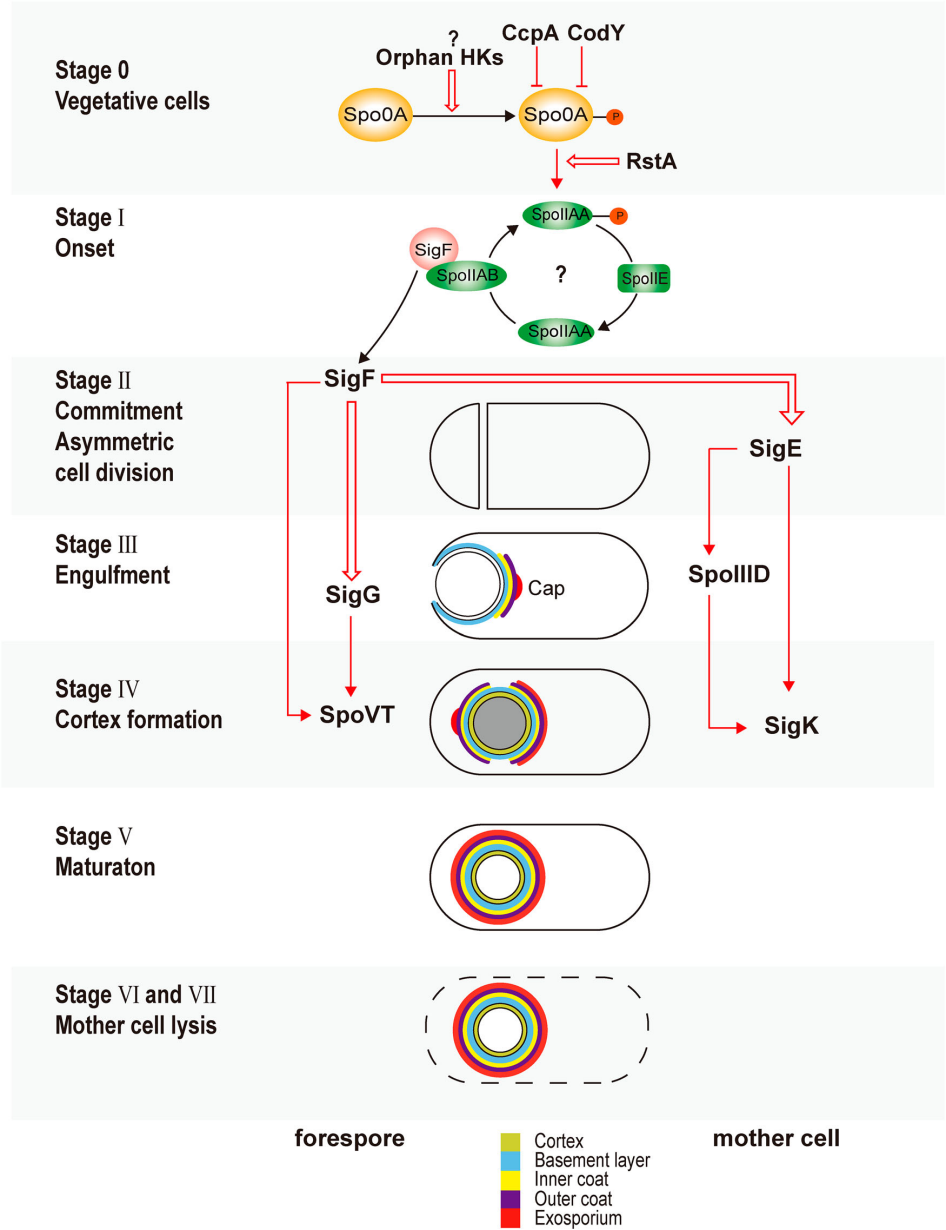
Sporulation of *Clostridioides difficile*. This figure summarizes the sporulation initiation and spore formation described in this review. Red arrows indicate transcriptional activation. Red cross arrows indicate transcriptional inhibition. Red bold arrows indicate post-translational regulations. Question marks indicate suggestive but unconfirmed mechanisms.

Once the initiation decision has been made, sporulation starts with a cascade of sporulation-specific RNA polymerase sigma factor activation [8,15,17,22,23]. As Figure 1 demonstrates, sporulation is arbitrarily divided into seven stages according to genetic and morphological changes [19]. At Stage 0, there is no obvious morphological change; however, the chromosome is replicated, which prepares the cell for sporulation. At Stage I, replicated DNA forms a single axial thread along the cell. At Stage II, a septum is formed at one end of the cell, which asymmetrically divides the cell into a larger mother cell and a smaller forespore. This is the first morphological stage of sporulation. At Stage III, the mother cell engulfs the forespore, which results in two membranes surrounding the forespore that are suspended in the cytosol of the mother cell. Coat proteins are orderly recruited to the proximal end of the forespore to form a layered cap. At Stage IV, a thick layer of modified peptidoglycan termed the cortex forms between the membranes surrounding the forespore, which confers the spore resistance to heat and ethanol [44]. At Stage V, coat proteins attached to the proximal pole spread along the surface of the forespore and encase it. The spore coat is composed of basement, inner, and outer layers, which function as a molecular sieve to protect the spore from enzymatic and chemical insults [19]. At stages VI and VII, the spore develops the final and outmost exosporium layer, which modulates the spore and environment interactions, followed by the lysing and releasing of the mature and metabolically inactive spore into the environment.

**Figure 2.**
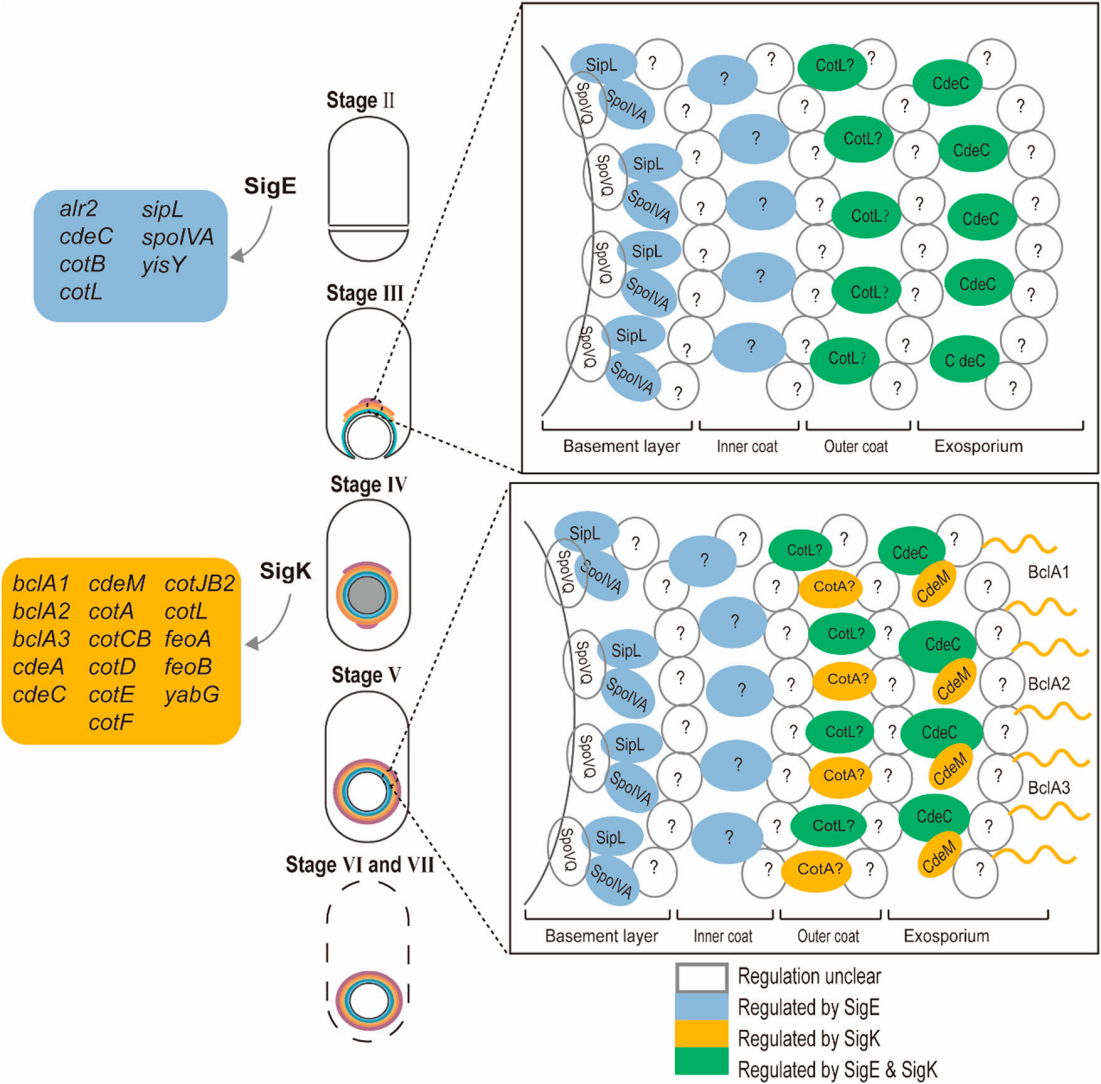
Coat assembly of *Clostridioides difficile.* This figure summarizes the coat assembly process described in this review. The coloured boxes on the left indicate SigE and SigK regulon. The box on the top right shows a proposed model of the spore coat assembly of the first stage, while the box on the bottom right shows the second stage. Question marks indicate unknown/unconfirmed proteins.

Coordinated with morphological changes of the spore, sigma factors, transcriptional factors, enzymes, and structural proteins are expressed in a timely order (Figure 1) [27,45–47]. After septum formation (Stage II), the sporulation continues in two different chambers as two separate, but connected, developmental lines, which are controlled by four sigma factors [15,17,48]. SigE and SigK control the development of the mother cell, while SigF and SigG control the forespore [17,48]. SigF is activated soon after septum formation (Stage II) in the forespore, possibly via the same mechanisms in *B. subtilis* that the anti–anti-sigma factor SpoIIAA is dephosphorylated by SpoIIE, and binds to the anti-sigma factor SpoIIAB [48]. Pro-SigE is processed to active SigE under the control of gene products regulated by SigF, via cross-septum interactions although SigF is not strictly required for the activation [15,27,45,46]. Following the activation of early sporulation sigma factors (SigE and SigF), the mother cell engulfs the forespore (Stage III) [15,48]. The activation of the late sporulation SigG in the forespore, however, happens before the completion of engulfment (Stage III), which is reliant on SigF but not on SigE, or at least less tightly regulated by SigE compared to its counterpart in *B. subtilis* [45,46,48]. SigF, SigG, and SpoVT form a feed-forward loop, such that both SigF and SigG are involved in the expression of SpoVT, whose mutant is able to complete the engulfment [46,49]. In contrast to SigG, the activation of SigK in the mother cell is after the engulfment (Stage IV), which is dependent on SigE, but not SigG [45,48]. SigE, SpoIIID, and SigK form a similar feed-forward loop to SigF, SigG, and SpoVT, respectively [46,50]. SigK controls the gene expression in the final stages of sporulation, including genes involved in the coat and exosporium assembly. The regulons of SigE, SigF, SigG, and SigK are determined through biochemical and transcriptomic research (Figure 2) [45,46].

## Spore coat assembly

The spore coat is a multi-layered, macromolecular, proteinaceous structure [8,19]. *C. difficile* spore coat has three distinct layers: the basement layer, inner coat, and outer coat (Figure 2) [19]. Similar to *B. subtilis*, the spore coat of *C. difficile* shows a relatively brighter and lamellated inner coat, and an electron-dense and striated outer coat under transmission electron microscopy (TEM) [17,19,51]. It confers the spore resistance to a wide range of chemical insults [8,19], probably through two mechanisms: first, it acts as a passive barrier that resists degradative enzymes and toxic molecules; second, it contains enzymes, catalases, for example, to detoxify the toxic molecules [19,52]. High-throughput studies have been performed to identify the proteins in the coat and exosporium of *C. difficile*, surprisingly, only ∼25% of coat proteins in *B. subtilis* are conserved in *C. difficile*, suggesting a very different assembly process [53,54].

Spore coat assembly is arguably the most complicated process in spore formation, which is driven by sequentially activated sigma factors and transcriptional factors [19,55]. The assembly of each layer of spore coat is dependent on one or two major morphogenetic proteins, as scaffolds to direct the recruitment of other proteins [15,56]. And the loss of the morphogenetic protein results in changes to the morphology and protein composition of the spore coat, and more importantly, reduction of spore yields and resistance to various insults. In *B. subtilis*, 10 morphogenetic proteins have been identified. A systematic study reveals that the basement layer is dependent on SpoIVA and SpoVM, the inner layer on SafA, the outer layer on CotE, and the crust on CotX, CotY, and CotZ [19,23,56]. On the contrary, only 7 morphogenetic proteins have been identified in the *C. difficile*, including SpoVQ, SpoIVA, SipL, CotL, CotA, CdeC, and CdeM (Figure 2) [51,57–59]. Among these proteins, SpoIVA and SipL localize to the basement layer and CdeC and CdeM to the exosporium layer (Figure 2). High-throughput methods are required to search for unidentified morphogenetic proteins in *C. difficile*. More importantly, the interacting networks need to be established among morphogenetic proteins, and between each morphogenetic protein and its cognate-binding proteins [60].

The first stage of assembly is the localization of coat proteins to form a layered cap on the mother cell proximal pole of the forespore (Figure 2, Stage III). In *B. subtilis*, the interactions of SpoVM and SpoIVA are required to anchor the basement layer to the forespore membrane [22,23]. SpoVM comprises an amphipathic helix, which recognizes the curvature of the forespore membrane, and embeds the hydrophobic side in the membrane. SpoIVA is the scaffold to direct the assembly of the basement layer of the cap [22,23]. Outer layers are localized sequentially to the mother-cell-proximal pole via protein–protein interaction, directed by SafA, CotE, and CotX/Y/Z, respectively [23]. In *C. difficile*, both SpoVM and SpoIVA are conserved, and SpoIVA functions similarly to its *B. subtilis* homologue [57,58,61,62]. SpoVM is confirmed to bind SpoIVA; however, about two-thirds of *ΔspoVM* forespores exhibit normal morphology, suggesting an insignificant role of SpoVM and a very different spore coat assembly mechanism in *C. difficle* [62,63]. Six more morphogenetic proteins, SpoVQ, SipL, CotA, CotL, CdeC, and CdeM, have been identified [51,52,58,64]. Nevertheless, the composition of each layer is unclear. Consequently, the physical interactive map of spore coat proteins of the first stage is yet to be drawn.

The second stage is known as the “encasement,” a morphological transition of the cap to spread and surround the circumference of the forespore (Figure 2, Stage III–V). And the encasement is accomplished by six distinct waves of protein localization in *B. subtilis*, according to a systematic study that tags ∼40 coat proteins with GFP to examine the time course of encasement [55]. The six waves of proteins are divided into two categories according to whether initial localization on the forespore surface occurred during or after engulfment. Proteins in the first category localize to the surface of the forespore before the completion of engulfment and form a full shell upon the completion of engulfment (Figure 2, stage III) [55]. The three interacting basement morphogenetic proteins, SpoVM, SpoIVA, and SpoVID, are included in the first category. The second category proteins, including the second to the sixth waves, polymerizes and/or proceeds around the circumference of the developing forespore after the engulfment, which requires a second nucleation site on the mother cell distal pole of the forespore (Figure 2, Stage IV). The mechanisms of encasement remain elusive; however, it is known that SpoVM and SpoVID are required for the encasement. One possible driving force of the encasement is the direct interactions of morphogenetic proteins. On the one hand, SpoIVA directly interacts with SpoVM, which recognizes and tracks the curvature of the forespore membrane during engulfment [65]; on the other hand, SpoVID functions as a bridge to link SpoIVA and other morphogenetic proteins, such that the N-terminal domain is required for the encasement, while the C-terminal region A directly interacts with SpoIVA [66,67]. Both SpoIVA and SpoVID recruit morphogenetic proteins in the outer layers of *B. subtilis* [63,68]. Another plausible driving force is the polymerization of morphogenetic proteins [23]. Consistent with this, SpoIVA polymerizes *in vitro* in an ATP-dependent manner, and SafA, CotE, and CotZ self-interact biochemically [23,69]. In *C. difficile*, SpoIVA is conserved and required for proper coat localization [57,62]. SpoVID has no homologue in *C. difficile*; however, SipL was identified via bioinformatic search to the C-terminal LysM domain of SpoVID, suggesting SipL is a functional homologue to *B. subtilis* SpoVID [57,58,61]. We then discuss *C. difficile* spore coat proteins that have been studied in the following part.

## SpoVM

Of the 10 spore coat morphogenetic proteins identified in *Bacillus subtilis*, only SpoVM and SpoIVA have homologues in *Clostridioides difficile* [18,19,23]. In *B. subtilis*, the basement layer is formed by the interactions of SpoVM, SpoIVA, and SpoVID. SpoVM is a 26 amino acid amphipathic helix, which embeds itself into the forespore membrane [18,22,23]. SpoVM interacts with the C-terminal region SpoIVA, which self-polymerizes with ATPase activity. SpoIVA binds to the C-terminal region A of SpoVID. Loss of SpoIVA results in no spore coat attached to the forespore. Disruption of either *spoVM* or *spoVID* blocks the encasement. In addition, loss of either SpoIVA or SpoVM inhibits cortex assembly, since checkpoint proteins, CmpA and SpoVID, inhibit the cortex assembly unless coat assembly initiates properly [66,70]. SpoVM plays a much less significant role in *C. difficile* sporulation in comparison to its homologue in *B. subtilis* sporulation [62]. *C. difficile* SpoVM binds to spoIVA; however, disruption of this interaction has relatively minor effects on spore formation. Loss of SpoVM exhibits a rather modest reduction in heat (∼3–4 fold) and chloroform (∼4 fold) resistance, in contrast to >6 log defects in *B. subtilis*, and results in only a ∼3-fold decrease of spore yield [62]. In addition, only ∼35% of *ΔspoVM* forespores exhibit morphogenetic abnormalities, including coat mislocalization, abnormal forespore shape, and variations in cortex thickness [62].

## SpoIVA and SipL

Both SpoIVA and SipL are morphogenetic proteins that are essential for the early development of spore coat [53,61]. SipL was identified in *C. difficile* through the bioinformatic search of the C-terminal LysM domain of SpoVID [61]. Only immature phase-dark spores were observed by phase-contrast microscopy for *ΔspoIVA* and *ΔsipL* mutant [61]. Either *ΔspoIVA* or *ΔsipL* mutant of *C. difficile* results in defects in spore coat localization [57,58,61]. Specifically, the spore coat is mislocalized to the mother cell cytosol, or only with a loose connection at the mother-cell-proximal pole of the forespore [61]. This phenotype resulted from the missing SpoIVA-SipL complex, which is essential for the spore coat assembly in *C. difficile* [58,61]. A closer examination reveals the LysM domain of SipL is responsible for the SpoIVA-SipL interaction [58,61]. The LysM domain may also directly bind to the peptidoglycan of the cortex [71]. Mutations in Walker-A ATP binding motif reduce the binding affinity, suggesting SipL binds to the ATP-bound form of SpoIVA [57]. However, the loss of either SpoIVA or SipL in *C. difficile* doesn’t affect the cortex formation, possibly due to the missing of the CmpA and SpoVID quality control [72].

## SpoVQ

SpoVQ is a morphogenetic protein and a binding partner to the SpoIVA-SipL complex via coimmunoprecipitation and mass spectrometry-based affinity proteomics [71]. Loss of *spoVQ* causes a 5-fold reduction in heat-resistant spore formation, and a 3-fold decrease in spore purification efficiency [71]. *spoVQ* mutant produces the cortex with only ∼60% thickness compared to the wild type and spore coat with similar thickness. Deletion of SpoVQ in either *ΔspoIVA* or *ΔsipL* mutant prevented cortex synthesis and impaired the localization of SipL to the forespore. Therefore, SpoVQ is proposed to function as a link between the cortex and coat assembly in *C. difficile*.

## CotL

CotL is a morphogenetic protein, even though proteomic studies couldn’t identify this protein, which may be due to the high lysine percentage [53,54]. However, CotL is detected in the coat/exosporium by western blotting and fluorescence microscopy [51]. *cotL* is expressed in the mother cell compartment and regulated by both SigE and SigK. Loss of CotL causes a significant reduction to the outmost layers (spore coat and exosporium) and a modest reduction to the cortex. Besides, the inactivation of *cotL* alters the abundance of proteins in the coat and exosporium layer, increases the permeability of the spore coat, and impairs the germination rate of spores [51]. CotL is proposed to localize between the early sporulation morphogenetic proteins and the exosporium morphogenetic proteins, considering the cotL mutant has a less severe defect in spore formation than the *spoIVA* or *sipL* mutant but a more severe phenotype than the cdeC mutant [51,57,58,61,64].

## CotA

CotA shares no homology with other proteins in the existing database, and is proposed to be a morphogenetic protein in *C. difficile* [52]. Three studies confirm CotA exposed on the surface of *C. difficile* spore, suggesting it localizes either in the outer spore coat or exosporium [52,73,74], though a recent study shows that intraperitoneally (i.p.) injection of CotA couldn’t protect mice from CD630Δerm strains [75]. Disruption of *cotA* results in a 74% and 71% reduction in colony-forming efficiency after being treated with lysozyme and ethanol, respectively, which is not observed in *cotB*, *cotCB*, *cotD*, *cotE*, or *sodA* mutants [52]. In addition, ∼50% of *cotA*::*CT555a* mutants showed a structural defect, such that the electron-dense outlayer of the spore coat was absent, leaving only the lamellated inner coat apparent [52]. A recent study suggests that the absence of CotA causes a disruption of the spore surface integrity, and consequently exposes and increases in binding sites of fidaxomicin [76]. Similar to CotL, the severity of *cotA* deletion is milder than *sipL* and *spoIVA* mutation, suggesting an outer localization in the spore coat.

## CotCB (CotJC1), CotD (CotJC2), and CotG

CotCB, CotD, and CotG are exposed on the surface of *C. difficile* spores, suggesting they are localized in either the outer spore coat or exosporium [52,73,74]. Disruption of *cotCB* or *cotD* doesn’t affect the spore yields, suggesting they are not morphogenetic proteins [52]. Bioinformatic analysis indicates they are putative manganese catalases [73]. CotCB and CotD are homologues, sharing ∼70% sequence similarity and ∼50% sequence similarity to their *B. subtilis* homolog CotJC [73]. In addition, CotG shares ∼35% of sequences with CotJC. Deletion of CotCB and CotD causes a reduction of catalase activity compared to WT *C. difficile* spores. Moreover, purified recombinant CotD and CotG are confirmed to carry catalase activity [52].

## CotE

CotE is localized in the outer spore coat or exosporium [52,73,74]. It is an 81-KD bifunctional protein, which carries chitinase activity at the carboxy-terminal domain and peroxiredoxin activity at the amino-terminal domain [52,77]. Loss of CotE in *C. difficile* results in no reduction of spore yield and germination rate, suggesting it doesn’t play a morphogenetic role [52,78]. On the contrary, CotE mediates the direct binding of spores to GlcNac and GalNAc in mucin glycoproteins and facilitates the degradation of mucin, which helps the host colonization of *C. difficile* [78]. Consistently, disruption of *cotE* increases the time, from colonization to clinical endpoint, and the survival rate of infected hamsters [78]. In addition, the *cotE* mutant shows a 1.3-fold decrease in fidaxomicin-bodipy binding, suggesting CotE could be a potential target for fidaxomicin binding [76].

## CdeC and CdeM

CdeC, 44.7 kDa and CdeM, 19.1 KDa, are two cysteine-rich proteins localized in the exosporium layer, which suggests a disulphide bridge formation and, therefore, a crosslinking role in exosporium [59,79]. Loss of CdeC, but not CdeM, affects *C. difficile* spore coat permeability, and, therefore, causes it more sensitive to ethanol and heating [64,79]. CdeC is conserved in a few Peptostreptococcaeace family members; however, CdeM is only identified in *C. difficile* [79]. Both CdeC and CdeM are regulated by sporulation-specific sigma factor K, suggesting they might be expressed during late sporulation stages, though there is a potential sigma E binding consensus in front of the cdeC gene [50,64,79]. CdeC and CdeM are both morphogenetic proteins in the exosporium layer, with CdeC playing a more important role. Inactivation of either CdeC or CdeM results in a reduction of the thickness of the exosporium layer and changes the relative abundance of the major protein species in both spore coat and exosporium [79]. However, A recent study shows that only CdeC, but not CdeM, is able to form organized inclusion bodies, filled with lamella-like structures with an interspace of 5–15 nm [59]. In addition, an increased oxidative environment or addition of DTT affects the self-organization ability, suggesting the organized structure is due to the formation of specific disulphide bonds which requires a subtle redox state [59]. Besides their morphogenetic role, CdeC and CdeM contribute to the adherence of *C. difficile* spores to the colonic mucosa [64,79]. Since they are exposed on the surface and antigenic, CdeM and CdeC, along with other surface proteins, have been utilized as immunization candidates [74,75].

## Future directions

Persistence and recurrence of CDI are suggested to be mediated by sporulation and biofilm formation. And the proteinaceous spore coat plays a key role in resistant chemical insults to protect *C. difficile* spore. Therefore, characterization of the dynamic process of spore coat assembly is essential to develop cures for recurrent CDI. However, a few key questions remain to be answered. The first question would be the composition of each layer of the spore coat. Morphogenetic proteins are scaffold proteins to direct the assembly of spore coat. Seven morphogenetic proteins have been identified in *C. difficile*, while ten were identified in *B. subtilis*. Considering the roughly equal number of spore coat proteins in *B. subtilis* and spore coat plus exosporium proteins in *C. diffficile*, it is reasonable to suspect there are more morphogenetic proteins yet to be recognized. Besides, it is confirmed that SpoIVA and SipL are localized to the basement layer, CdeC and CdeM in the exosporium, but CotL and CotA remained unclear. Therefore, high-throughput methods need to be developed to characterize the interactions among morphogenetic proteins, and between morphogenetic proteins and their binding partners. This information is the direct guidance to develop inhibitors against *C. difficile* spore coat assembly.

Besides the composition and interaction of each layer, the second question to be answered is the time and order of proteins recruited to the forespore. Spore coat proteins are categorized into two classes according to the encasement of protein during or after the engulfment. SpoVM and SpoVID are required for successful encasement in *B. subtilis*. However, SpoVM plays a much less significant role in *C. difficile*. And the function of SpoVID seems to be replaced by SipL. Therefore, systematic studies are in need to characterize the encasement waves.

The third question is the link between the cortex and coat assembly. In *B. subtilis*, they are coordinated through the interactions of SpoVM, SpoIVA, and SpoVID, and through the quality control of CmpA and SpoVID. This co-assembly mechanism is not yet identified in *C. difficile*, at least is not related to the known morphogenetic proteins SpoIVA and SipL. The recently identified SpoVQ might play a role to link these two processes.
